# Supplementary material for: Time-series transcriptome analysis identified differentially expressed genes in broiler chicken infected with mixed Eimeria species
Source: Front Genet. 2022 Aug 8;13:886781. doi: 10.3389/fgene.2022.886781 (PMC9393255; doi:10.3389/fgene.2022.886781)
Supplement: Supplementary file 2 [file DataSheet1.ZIP › 7dpi_GO.Gsea.1625106495923/GOMF_NAD_P_H_DEHYDROGENASE_QUINONE_ACTIVITY.html]

Details for gene set GOMF\_NAD\_P\_H\_DEHYDROGENASE\_QUINONE\_ACTIVITY[GSEA]

|  || Dataset | TMM\_7dpi\_gct\_format\_7dpi\_gct\_format.Class\_4dpi.cls #PC\_versus\_NC.Class\_4dpi.cls #PC\_versus\_NC\_repos |
| Phenotype | Class\_4dpi.cls#PC\_versus\_NC\_repos |
| Upregulated in class | 1 |
| GeneSet | GOMF\_NAD\_P\_H\_DEHYDROGENASE\_QUINONE\_ACTIVITY |
| Enrichment Score (ES) | 0.7903864 |
| Normalized Enrichment Score (NES) | 3.291886 |
| Nominal p-value | 0.0 |
| FDR q-value | 0.0 |
| FWER p-Value | 0.0 |
Table: GSEA Results Summary

  

Fig 1: Enrichment plot: GOMF\_NAD\_P\_H\_DEHYDROGENASE\_QUINONE\_ACTIVITY      
 Profile of the Running ES Score & Positions of GeneSet Members on the Rank Ordered List

  

| SYMBOL | TITLE | RANK IN GENE LIST | RANK METRIC SCORE | RUNNING ES | CORE ENRICHMENT || 1 | NQO1 | na | 9 | 1.855 | 0.0864 | Yes |
| 2 | NDUFA2 | na | 303 | 0.935 | 0.1058 | Yes |
| 3 | NDUFB6 | na | 307 | 0.933 | 0.1494 | Yes |
| 4 | NDUFS5 | na | 308 | 0.932 | 0.1932 | Yes |
| 5 | NDUFB7 | na | 323 | 0.915 | 0.2350 | Yes |
| 6 | NDUFB2 | na | 348 | 0.885 | 0.2746 | Yes |
| 7 | NDUFAB1 | na | 356 | 0.876 | 0.3152 | Yes |
| 8 | NDUFA7 | na | 371 | 0.857 | 0.3542 | Yes |
| 9 | NDUFC2 | na | 394 | 0.834 | 0.3916 | Yes |
| 10 | NDUFA13 | na | 557 | 0.708 | 0.4113 | Yes |
| 11 | NDUFS3 | na | 623 | 0.675 | 0.4375 | Yes |
| 12 | NDUFB3 | na | 656 | 0.657 | 0.4657 | Yes |
| 13 | NDUFS8 | na | 661 | 0.653 | 0.4961 | Yes |
| 14 | NDUFS6 | na | 686 | 0.642 | 0.5242 | Yes |
| 15 | NDUFA1 | na | 742 | 0.616 | 0.5486 | Yes |
| 16 | NDUFA5 | na | 794 | 0.593 | 0.5722 | Yes |
| 17 | NDUFB5 | na | 902 | 0.554 | 0.5893 | Yes |
| 18 | NDUFV3 | na | 988 | 0.527 | 0.6069 | Yes |
| 19 | NDUFB4 | na | 1015 | 0.517 | 0.6290 | Yes |
| 20 | NDUFA12 | na | 1023 | 0.513 | 0.6526 | Yes |
| 21 | NDUFA6 | na | 1079 | 0.492 | 0.6711 | Yes |
| 22 | NDUFA8 | na | 1080 | 0.492 | 0.6942 | Yes |
| 23 | NDUFS2 | na | 1124 | 0.477 | 0.7130 | Yes |
| 24 | NDUFB10 | na | 1215 | 0.450 | 0.7266 | Yes |
| 25 | NDUFV1 | na | 1307 | 0.428 | 0.7391 | Yes |
| 26 | NDUFB8 | na | 1330 | 0.423 | 0.7571 | Yes |
| 27 | NDUFS7 | na | 1371 | 0.412 | 0.7732 | Yes |
| 28 | NDUFV2 | na | 1395 | 0.408 | 0.7904 | Yes |
| 29 | NDUFB9 | na | 1842 | 0.299 | 0.7672 | No |
| 30 | NDUFB1 | na | 1995 | 0.272 | 0.7673 | No |
| 31 | NQO2 | na | 2041 | 0.264 | 0.7759 | No |
| 32 | NDUFA4 | na | 2726 | 0.160 | 0.7263 | No |
| 33 | NDUFA9 | na | 2879 | 0.141 | 0.7202 | No |
| 34 | NDUFA10 | na | 3412 | 0.073 | 0.6792 | No |
| 35 | NDUFS1 | na | 3763 | 0.033 | 0.6515 | No |
| 36 | NDUFS4 | na | 4402 | -0.040 | 0.6001 | No |
| 37 | CBR4 | na | 6527 | -0.257 | 0.4348 | No |
| 38 | NDUFAF2 | na | 8545 | -0.491 | 0.2894 | No |
Table: GSEA details [plain text format]

  

Fig 2: GOMF\_NAD\_P\_H\_DEHYDROGENASE\_QUINONE\_ACTIVITY      
 Blue-Pink O' Gram in the Space of the Analyzed GeneSet

  

Fig 3: GOMF\_NAD\_P\_H\_DEHYDROGENASE\_QUINONE\_ACTIVITY: Random ES distribution      
 Gene set null distribution of ES for **GOMF\_NAD\_P\_H\_DEHYDROGENASE\_QUINONE\_ACTIVITY**
